# A case report of pregnancy and paliperidone palmitate 3‐monthly long‐acting injection

**DOI:** 10.1002/ccr3.3213

**Published:** 2020-08-11

**Authors:** Rita de Azevedo Avelar, Antonio Luengo Corbal, Maria João Heitor

**Affiliations:** ^1^ Psychiatry and Mental Health Department Hospital Beatriz Ângelo Loures Portugal

**Keywords:** paliperidone, pharmacology, pregnancy, psychiatry, schizophrenia

## Abstract

This clinical case reports the use of paliperidone palmitate 3‐monthly long‐acting injection during pregnancy without known adverse effects on the baby's development.

## Graphical abstract

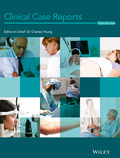

## 1 INTRODUCTION

Paliperidone is a metabolite of risperidone, a second‐generation antipsychotic drug commonly used nowadays. It was approved in Europe in 2007 and its long‐acting injectable solution in 2011. Paliperidone palmitate 3‐monthly long‐acting injection is an option as maintenance treatment for schizophrenia in Europe since 2014 and is as effective as the monthly injection.^1^ It has been used in adults who have been stable with paliperidone once‐monthly injections treatment. Recent data suggest that there is an increase in conception rates of women with psychotic disorders and taking antipsychotics.^2^ There is also evidence showing that women with psychotic disorders tend to have unplanned pregnancies more frequently.^3^ Safety studies of pharmacological substances during pregnancy are not allowed and information on the use of paliperidone in pregnancy is scarce, specifically, two published clinical cases and a database analysis.^4^, ^5^, ^6^ This case study reports the use of paliperidone palmitate 3‐monthly long‐acting injection in a pregnant woman, diagnosed with schizophrenia, who gave birth to a healthy newborn.

## 2 CASE REPORT

A 26‐year‐old caucasian female comes to the emergency room complaining about abdominal pain. After examination, it became evident that she was pregnant and labor had started. The pregnancy was not planned nor monitored. Regarding her medical background, she was diagnosed with schizophrenia at the age of 20, when she was hospitalized in the context of bizarre and paranoid delusional ideas, perplexity, disorganized speech, loosening of associations, inappropriate laughs, and total absence of insight. No other medical comorbidities were found. By that time, she was consuming cannabinoids daily, a habit she abandoned after her first psychotic episode. There is no record on the use of other drugs. Regarding family history, her mother had depressive disorder and a cousin had schizophrenia. During this hospitalization, she was firstly treated with olanzapine. However, since there was no improvement, a switch was made to clozapine, titrated until 600 mg, then reduced to 400 mg, due to transaminases elevation and fever. After 36 days of treatment she was discharged and, on her first outpatient consultation, clozapine was reduced to 200 mg, due to weight gain, somnolence, and amenorrhea. Subsequently, she began to miss follow‐up appointments, having reduced clozapine by herself. In the following consultation, a change to risperidone 6mg was made, as she maintained marked weight gain and amenorrhea, with high risk of therapeutic noncompliance. She abandoned psychiatric follow‐up and, approximately 10 months later, she was brought to the hospital due to dysphoric mood, evasive posture, and hostile contact, and it was found that she had abandoned antipsychotic therapy. At that stage treatment with risperidone was resumed and 100 mg of paliperidone palmitate monthly injection was added. The patient complied with this treatment for 3 months, when she abandoned monitoring and medication, and fled to another city. She was hospitalized three months later, presenting with aggressive contact, irritable mood, and disorganized behavior. She was discharged to compulsory outpatient treatment, on 6mg risperidone, which she abandoned, and monthly 75 mg paliperidone palmitate. As previously mentioned, during this process she suffered noticeable weight gain and amenorrhea, that lasted several months, attributable to the antipsychotic therapy. These side effects might have contributed to the underestimation of eventual signs of pregnancy. During one and a half year, she continued the injectable medication and risperidone was gradually stopped. After that time, the doctor considered to stop the injectable medication and started paliperidone 9 mg daily. However, one year later, psychotic symptoms arose and, according to her history of poor compliance with medication it was necessary to restart a long‐acting drug. Palmitate paliperidone monthly long‐acting injection 100 mg was prescribed, together with quetiapine 300 mg, for emotional instability, irritability, and insomnia. After one and a half year, quetiapine had been stopped and palmitate paliperidone monthly long‐acting injection was reduced to 75 mg. After a total of 17 monthly injections of palmitate paliperidone, either taken at the hospital or in the community to ensure compliance, a switch was made to palmitate paliperidone 3‐monthly long‐acting injection 263 mg, to facilitate treatment maintenance in a patient prone to avoid it and requiring a lot of family effort for compliance. The 3‐monthly injection was administered twice, the last one approximately 2 months before the infant's birth. She was on no other medication. When she arrived at the emergency service, she had no psychotic symptoms, was in a relationship and had a job, although it should be mentioned that the patient has mild cognitive deficit which led to school failure and difficulty in obtaining a job and a driver's license. In the emergency room, she complained of abdominal pain and reported she had noticed abdominal bloating, which she did not value. After medical examination, pregnancy was considered although she had amenorrhea that lasted more than 1 year. On gynecological examination, a vaginal discharge of white fluid was noted, which according to the patient had started earlier that day. The echography showed the presence of a fetus in cephalic position, with cardiac sounds present. Since the pregnancy was not monitored and the position of the fetus prevented the measurement of biometrics, it was not possible to calculate how many weeks it lasted. Fetal morphological ultrasound, cardiotocography, or laboratorial studies were not performed during the entire pregnancy. She was taken to the delivery room and gave birth to a healthy newborn male with 2420grams, with an APGAR score of 9‐10‐10, without complications. The patient and the family were pleased with the baby's arrival. Social care was involved in social risk evaluation, to guarantee the protection of the baby. He was closely monitored, and there were no signs or symptoms during the first 2 weeks after delivery. The mother decided not to breastfeed. To the date of this case report, approximately 1 year after birth, the child showed no health or developmental issues. With regard to the patient's follow‐up, she continues to receive palmitate paliperidone 3‐monthly injection and is asymptomatic.

## 3 DISCUSSION

Antipsychotic use during pregnancy is a concern that clinicians often have to deal. All antipsychotics cross the placenta with potential risk to the fetus. In this case report, all the antipsychotics the patient was medicated with, with exception of clozapine, fall into the category C or D, meaning that the risk to the fetus cannot be ruled out or that there is evidence of human risk, respectively.^7^ Animal studies demonstrated that paliperidone palmitate, injected intramuscularly or orally given, have not been found to be teratogenic, although reproductive toxicity has been detected. ^8^, ^9^ It is also stated that after a single dose of 3‐monthly long‐acting injection of paliperidone palmitate, paliperidone will be detectable in plasma up to 18 months, which should alert the clinician to possible effects on the newborn.^9^ It has been reported that neonates exposed to paliperidone during the last trimester of pregnancy have a higher probability of showing extrapyramidal or withdrawal symptoms after delivery.^8^, ^9^ The precursor of paliperidone, risperidone, was associated with an increased risk of malformations during the first trimester of pregnancy and therefore recommending further studies.^10^ A systematic review showed a diminished gestational age of approximately 37 weeks in women medicated with second‐generation antipsychotics during pregnancy but birth weight was within normal range.^11^ In this case report, the pregnancy was undetected until delivery, so it was not possible to calculate the duration of the gestation. The newborn had 2420grams, just under 2500grams, the lower range of adequate weight according to the World Health Organization standards.^12^ Breastfeeding is not advised as the molecule is excreted in milk.^8^, ^9^ In the literature, two case reports have been published on the use of palmitate paliperidone monthly long‐acting formulation during pregnancy and one on the use of risperidone long‐acting injection, the precursor of paliperidone.^5^, ^6^, ^13^ Similarly, no adverse effects were found. Newborns should be closely monitored after delivery since there have been reports of agitation, sedation, tremor, muscular tone alteration, and feeding issues when the mother was treated with antipsychotics during pregnancy.^8^, ^9^ Electroconvulsivotherapy is considered a safe option when dealing with a pregnant woman who presents with psychotic symptoms. ^7^

## 4 CONCLUSION

To our knowledge, this is apparently the first case report on the use of paliperidone palmitate 3‐monthly long‐acting injection in a pregnant woman, diagnosed with schizophrenia, who gave birth to a baby where no health or neurodevelopmental problems were observed. The decision of maintaining antipsychotic medication during pregnancy should take into account a risk‐benefit analysis. In some cases, the interruption of treatment may represent an important risk of relapse of psychiatric illness, compromising the wellbeing, health, and care of the baby.

## CONFLICT OF INTEREST

The authors declare no conflict of interests, nor affiliation with or involvement in any organization or entity with financial or nonfinancial interest.

## AUTHOR CONTRIBUTIONS

RA evaluated the patient during the postpartum period and presented the idea. AC is the doctor who has been following the patient since her first psychiatric hospitalization and encouraged the investigation about the theme, which was performed by RA. MJH supervised the findings of this work. All authors discussed the clinical case and contributed to the final manuscript.

## ETHICAL APPROVAL

This clinical case report was approved by the ethical committee of Hospital Beatriz Ângelo. The patient gave her informed consent. This research was conducted under the Declaration of Helsinki code of ethics.
